# A Genetic Assay for Transcription Errors Reveals Multilayer Control of RNA Polymerase II Fidelity

**DOI:** 10.1371/journal.pgen.1004532

**Published:** 2014-09-18

**Authors:** Jordan D. Irvin, Maria L. Kireeva, Deanna R. Gotte, Brenda K. Shafer, Ingold Huang, Mikhail Kashlev, Jeffrey N. Strathern

**Affiliations:** 1 NCI Center for Cancer Research, Frederick, Maryland, United States of America; 2 U.S. Army Medical Research and Materiel Command, Fort Detrick, Maryland, United States of America; University of Wisconsin-Madison, United States of America

## Abstract

We developed a highly sensitive assay to detect transcription errors *in vivo*. The assay is based on suppression of a missense mutation in the active site tyrosine in the Cre recombinase. Because Cre acts as tetramer, background from translation errors are negligible. Functional Cre resulting from rare transcription errors that restore the tyrosine codon can be detected by Cre-dependent rearrangement of reporter genes. Hence, transient transcription errors are captured as stable genetic changes. We used this Cre-based reporter to screen for mutations of *Saccharomyces cerevisiae RPB1 (RPO21)* that increase the level of misincorporation during transcription. The mutations are in three domains of Rpb1, the trigger loop, the bridge helix, and in sites involved in binding to TFIIS. Biochemical characterization demonstrates that these variants have elevated misincorporation, and/or ability to extend mispaired bases, or defects in TFIIS mediated editing.

## Author Summary

Mistakes made during the synthesis of messenger RNAs have been difficult to detect, both because mRNAs can be short lived, and because the translation of mRNAs into proteins has a much higher error rate that masks transcription errors. We present here a highly sensitive genetic screen that detects transcription errors and use it to identify mutations that increase the error rate of RNA polymerase II. The screen incorporates a new principle that allows transient transcription errors to cause permanent genetic changes. The screen is based on suppression of a missense mutation (*cre-Y324C*) in the active site of the Cre recombinase. Infrequent and transient transcription errors that restore the original codon for Y324 cause the Cre-dependent activation of a reporter gene. Background from translation errors is negligible because Cre acts as a tetramer in which all four subunits require the active site tyrosine. Transcription errors as low as ∼10^−6^ can be detected. We identify *rpb1* mutations that define four classes, those that have increased (1) misincorporation, (2) extension of a misincorporated base, (3) both misincorporation and extension, and (4) those that block the activity of the transcription proofreading factor, TFIIS.

## Introduction

Accurate transcription is an essential step in accessing the genetic information stored in genes. In eukaryotes, transcription of DNA into mRNA is carried out by RNA polymerase II (Pol II), an enzyme comprised of 12 subunits. While there has been extensive research on core Pol II, including biochemical and detailed structural information [1]–[7], less is known about how the accuracy of transcription is controlled. The net misincorporation rate is estimated to be about one per 10^5^ bases [8], [9]. This reflects initial misincorporation by Pol II and subsequent editing mechanisms, some intrinsic to the core polymerase and others facilitated by transcription factors. Direct screens for mutations that reduce the fidelity of transcription have been difficult due to the transient nature of the errors and the relatively high rate of translation errors, particularly at nonsense codons, that can mask transcription errors [8], [10]–[12]. Here we report a novel approach to identifying mutations that increase transcription errors *in vivo*.

Rpb1 and Rpb2 are the two largest Pol II subunits with several structural and functional domains that are implicated in transcription fidelity maintenance. Rpb1 contains the active site for nucleotide addition, and, together with Rpb2, forms a substrate-binding site and a deep channel accommodating template DNA and a 9–10 bp RNA-DNA hybrid [13]. Mutations in the RNA-DNA hybrid binding cleft or near the substrate binding site cause increased occurrence of insertions and deletions during transcription through homopolymeric tracts [14]–[16]. A mutation that appeared to reduce the accuracy of transcription was identified in *rpoB*, the gene coding for second largest subunit of RNA polymerase in *Escherichia. coli*, among rifampicin resistant mutants [8], [17]. The molecular mechanism of fidelity maintenance disrupted by this mutation remains to be established. However, several mechanisms by which Rpb1 regulates fidelity have been elucidated.

Important structural elements surrounding the Pol II active site include the Rpb1 bridge helix, separating the active site from the downstream DNA binding channel, and the trigger loop, a mobile element of Rpb1, which undergoes dramatic conformational changes during each NTP addition [7], [18]. The trigger loop opens allowing for Pol II translocation and NTP entry to the active site, and closes, interacting with the substrate NTP and promoting catalysis. Mutations in the trigger loop that increase incorporation of non-complementary substrates and dNTPs and thus decrease Pol II fidelity *in vitro* have been identified from secondary screens of Pol II mutants [3], [19], [20]. Rpb1 interacts with non-essential Pol II subunit Rpb9, which is implicated in transcription fidelity maintenance [11], [21]–[24]. It is likely that Rpb9 prevents misincorporation indirectly, by attenuating the trigger loop closing [23]. Rpb9 also has been shown to prevent extension of the misincorporated base [24]. Transcription elongation factor TFIIS interacts with Pol II Rpb1 and is implicated in fidelity control at post-incorporation stage [12], [25], [26]. Indeed, it has been demonstrated that *in vitro* TFIIS preferentially promotes cleavage of the mismatched 3′end of nascent RNA [27].

The interpretation of phenotypic changes associated with alterations in RNA polymerase is complicated by a mix of direct and indirect changes. RNA polymerase mutations can change the transcriptome by altering the initiation differentially at promoters, by changing elongation rates, which in turn can alter splicing efficiency, and by altering termination [28], [29]. To focus on the identification of transcription fidelity mutants, we combined a primary genetic screen for transcription errors with a biochemical assays to identify the nature of the transcription fidelity defect. In summary, mutations in Rpb1 trigger loop rendering Pol II error-prone, as well as mutations in TFIIS binding site and deletions of *RPB9* and *DST1* (the gene encoding TFIIS) are demonstrated to reduce the fidelity of transcription *in vivo*.

## Results

### Transcription fidelity assay based on Cre recombinase

Assays of transcription fidelity based on nonsense or missense suppression are problematic in part due to the high background caused by translational errors [10]–[12], [30]. In the approach reported here, we rely on a requirement for an active tetramer to reduce the contribution of translation errors on the background. In addition, previous approaches have required a continual production of transcription errors to produce the monitored phenotype. In our approach, transient transcription errors can result in a stable genetic change. The system involves three parts, 1) a missense mutation in the active site tyrosine of Cre recombinase, 2) a Cre-dependent recombination reporter substrate with a very low Cre-independent rate of recombination and 3) promoters that give low level expression of the mutant *cre* allele. These three aspects of the system are discussed separately below.

### Cre mutations

**Figure 1:**
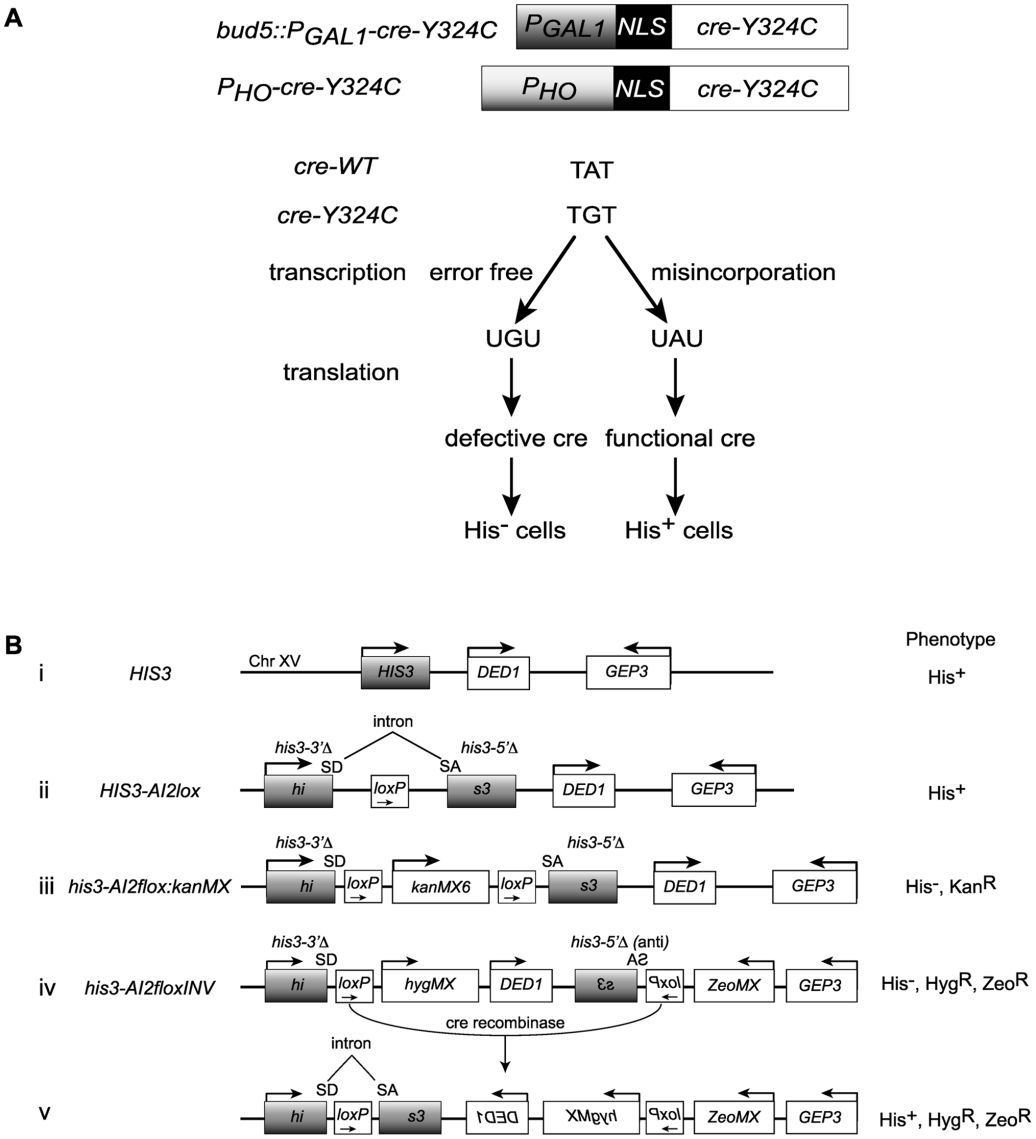
Cre-recombinase based assay for transcription errors *in vivo*. (**A**) Two constructs based on the promoters for the *GAL1* and *HO* genes that allow low level expression of a mutant variant of the Cre recombinase (*cre-Y324C*) that has a cysteine substitution for the active site tyrosine. Suppression of the A to G mutation can result from transcriptional misincorporation of an A at that position yielding transient Cre activity. (**B**) Transient Cre activity can be detected as recombination events that restore function to a *HIS3* based reporter. (i) The interval near the normal *HIS3* gene. (ii) A loxP site inserted into an artificial intron in *HIS3* is still a functional gene. SD = splice donor, SA = splice acceptor. (iii) Insertion of the *kanMX* gene flanked by loxP sites into the intron results in loss of *HIS3* function. (iv) Inversion of the C-terminal portion of the *his3* gene renders it defective. (v) Cre-mediated inversion of the construct in iv results in a functional *HIS3* gene.

Cre functions as a homotetramer, recognizing two 34 bp DNA sequences termed loxP and promoting recombination of their flanking DNAs [31], [32]. Depending on the orientation of the two loxP sites, the flanking DNA is either inverted (intrachromosomal antiparallel), excised (intrachromosomal direct), or exchanged (interchromosomal) [33]. The active site tyrosine (Y324) of Cre recombinase is essential for its activity [34]. All four subunits of the tetramer become covalently attached to strands of the DNA via those tyrosine 324 side chains during the recombination reaction. Chimeric enzymes assembled from mutant and WT subunits are catalytically inactive [35]–[39]. Translation errors that produce a WT monomer will not result in active Cre, whereas a transcription error that restores the WT codon can be translated into multiple WT subunits and assembled into an active Cre tetramer. The experiments presented here focus on the Y324C allele. This TAT → TGT mutation requires Pol II to misincorporate an adenosine opposite a cytosine in the template strand in order to suppress the *cre-Y324C* defect (Figure 1A). This G → A transcription error is among the most common mistakes made by *E. coli* RNAP *in vitro* and *in vivo* [40] and by *S. cerevisiae* [19].

### Cre reporters

**Figure 2:**
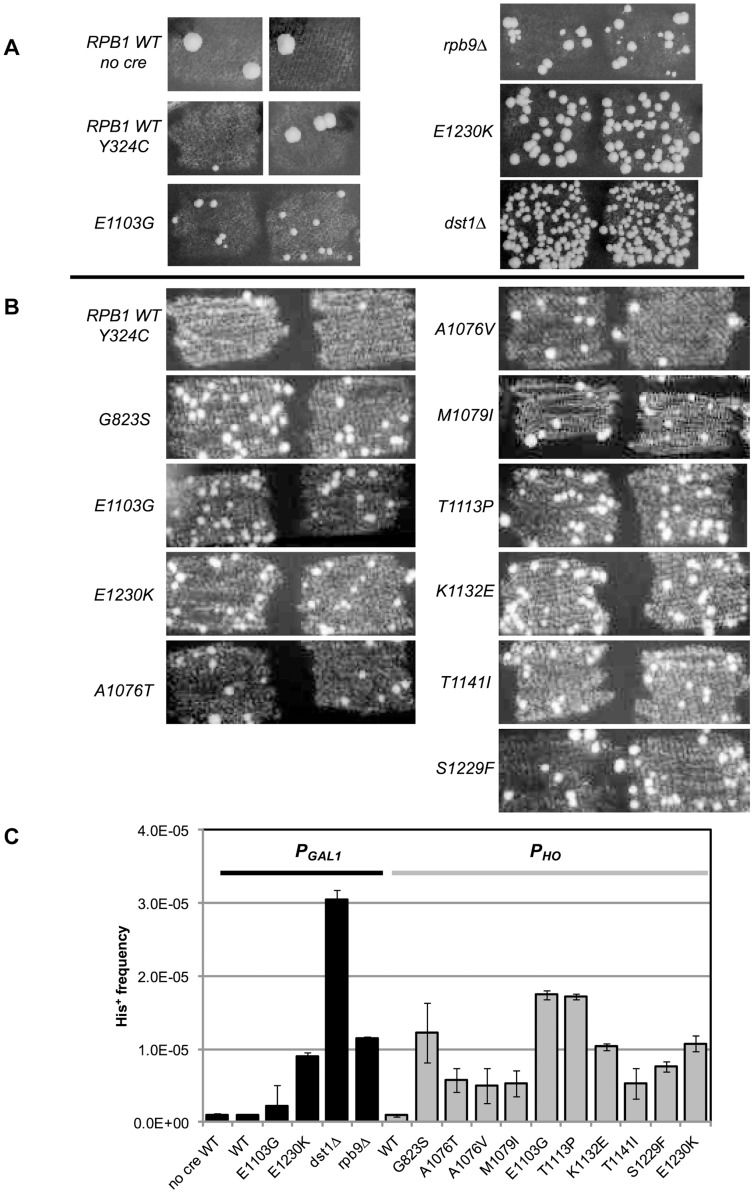
Suppression of *cre-Y324C* is elevated in strains with Pol II fidelity defects. (**A**) *P_GAL1_-cre-Y324C* strains grown in glucose on plates lacking histidine show increased numbers of cells capable of growing microcolonies in strains with known transcription fidelity defects. Two representative patches are shown for each variant. (**B**) *P_HO_-cre-Y324C* used to identify new transcription fidelity mutants. (**C**) Mean frequency of His^+^ cells relative to total viable cells in *P_GAL1_-creY324C*, or *P_HO_-creY324C*, *his3-AI2floxINV* strains with Pol II mutants. Cultures were grown overnight in 1 ml of YPD at 30°C and plated on SC-His and 10-fold serial dilutions onto YEPDA plates. Plates were incubated at 30°C for 3 days and individual colonies were scored. Error bars show standard error.

We created a reporter for Cre activity based on *HIS3* into which we placed an artificial intron [41] carrying a loxP site, *HIS3-AI2lox* (Figure 1B, construct (ii)). A Cre activatable derivative, *his3-AI2floxMX*, was made by insertion of a *kanMX* cassette (Figure 1B, construct (iii)) [42], [43]. However, the Cre-independent His^+^ background from this reporter was too high (∼2×10^−6^ His+ among total cells). We created a *HIS3* based Cre reporter system with very low Cre-independent background by placing the downstream loxP site, splice acceptor and C-terminal portion of a *his3-AI2floxMX* reporter in an inverted orientation at a position 3 kb distal of *HIS3* beyond the *DED1* gene (Figure 1B, construct (iv)). The N-terminal portion of *his3-AIlox* is at the normal chromosome XV position for *HIS3* and is marked by *hygMX*, while the C-terminal portion of *his3-AIlox* was marked with a *zeoMX* cassette. Cells with this inverted lox reporter, designated *his3-AI2floxINV*, are His^−^, hygromycin resistant and zeomycin resistant. If Cre is active, it can cause inversion of the interval between the two loxP sites to generate the functional *HIS3-AI2lox* gene (Figure 1B, construct (v)). These cells are His^+^, hygromycin resistant and zeomycin resistant. The frequency of Cre-independent His^+^ cells among total cells with this system is <10^−6^ (Figure 2C).

### Low level expression

In the experiments described here it was anticipated that the mutant Cre-Y324C protein should act as a dominant inhibitor of wild type Cre protein. That is, assembly of mutant subunits into the tetramer will block its function. Therefore, we expected that it is important to have very low levels of expression in order to favor assembly of tetramers from translation of one mRNA. This condition promotes the detection of transcription errors that restore the Tyr codon in the *cre-Y324C* transcript. We used two approaches to have low levels of transcription (Figure 1A). First we placed the *cre* gene under the control of the *GAL1* promoter but grew the cells in the presence of glucose. Under these glucose repressed conditions, expression was expected to be rare. In the second approach, we placed the *cre-Y324C* allele under the control of the *HO* promoter so that it would be expressed only in that half of the cell population that had already divided once before [44], [45].

### Proof of principle

Our initial experiments used the *P_GAL1_-cre-Y324C* expression system as the transcription error substrate (inserted into the *BUD5* gene) and the *his3-AI2floxINV* Cre activity reporter. For cells with a WT Pol II (GRY3739) the frequency of His^+^ cells among the total population is similar in the absence of Cre or with the *P_GAL1_-cre-Y324C* construct (Figure 2A&C). In this assay, patches of cells grown on rich media (YPD) were replica plated onto synthetic complete medium lacking histidine. The starting strains cannot grow, but rare His^+^ cells within the patch can grow into small colonies or papillae. The *P_GAL1_-cre-Y324C* strain lacking TFIIS (*dst1Δ* GRY3742) has an elevated level of His^+^ cells (Figure 2A&C) that is not observed when the *cre* gene is absent. We interpret the elevated level of papillation in the TFIIS defective strain as a reflection of transient Cre activity caused by uncorrected G → A errors during transcription in the mutant *cre-Y324C* gene that restore the UAU tyrosine codon in the mRNA (Figure 1A). To demonstrate that the His^+^ cells were not a result of an elevated reversion rate for the *cre-Y324C* mutation in TFIIS defective cells, 11 independent His^+^ cells from the *dst1* strain were crossed to a cell carrying an *ade6-AI2floxkanMX* reporter and shown to be still defective for Cre recombinase activity. In addition, the *cre* gene sequenced from 12 more His^+^ colonies still carried the *cre-Y324C* mutation. To determine whether the increase in His^+^ cells might be the result of elevated Cre-independent recombination between the lox sites in TFIIS defective strains, we compared the frequency of His^+^ cells in the absence of the *cre* gene and observed that WT and *dst1* strains gave similar levels. These results confirm that TFIIS has a role *in vivo* in editing RNA errors and promoting transcription fidelity.

*RPB9* has been identified as a nonessential subunit of Pol II that promotes accurate start site selection, efficient elongation, contributes to transcription fidelity [11], [23], [46]–[50] and regulates mismatch extension [24]. Consistent with a role for the Rpb9 subunit in promoting transcription fidelity *in vivo*, we find that a null allele of *RPB9*, *rpb9-Δ0* (GRY3743), shows an elevated His^+^ papillation rate in the *his3-AI2floxINV* assay (Figure 2A&C).

In order to test a collection of *rpb1* mutations, we created a strain (GRY2855) with the *P_GAL1_-cre-Y324C* substrate, the *his3-AI2floxINV* reporter and the *rpb1-natMX* null allele complemented by *RPB1* on a *URA3* selectable plasmid (pJDI220). We made *rpb1* variants on a *LEU2* based plasmid (pJS757) and substituted the mutant alleles for the *RPB1* wild type allele by transforming in the *LEU2* plasmids and selecting for loss of the *URA3* plasmid with 5-FOA which selects against Ura^+^ cells [51]. Cells with the *rpb1-E1230K* (*rpo21-24*) mutation (pJS932), which blocks the ability of TFIIS to bind to Pol II [52], show an elevated number of His^+^ papillae (Figure 2A&C). This is consistent with the results presented above for the TFIIS defective strain (*dst1Δ0*). We previously described *rpb1-E1103G* (pJS781) as causing increased misincorporation during transcription [19]. That allele was also among those identified as having elevated misincorporation rates *in vitro* [3]. When put into the *his3-AI2floxINV* reporter strain with *cre-Y324C* under the control of the *GAL1* promoter, it showed a modest increase in His^+^ papillation (Figure 2A&C).

### Genetic screen for new *rpb1* mutants

To use the *his3-AI2floxINV* reporter as an effective screen for new *rpb1* mutations that reduce fidelity, we addressed the problem of how to get widespread but low level expression of the *cre-Y324C* gene. The ideal condition would be the one that produced one transcript per cell. The *GAL1* promoter in the presence of glucose is repressed [53], but low levels of transcription in a population probably reflects some cells with transcripts and most cells with none. The promoter for the *HO* gene is under complex regulation so that it is only expressed late in the G1 phase of the cell cycle, only in cells that have the **a** or α mating phenotypes (not **a**/α “diploid” cells), and only in cells that have divided at least once before, so called mother cells [44], [45]. We placed the *cre-Y324C* gene under the control of the *HO* promoter at its normal position on chromosome IV. Only half of the cells (mothers) in the population will be expressing *P_HO_-cre-Y324C*, and half of those will be expressing it for the first time. We reasoned that this would create a condition where rare transcripts that have an error that restores the tyrosine codon would produce active Cre recombinase that would not be in competition with the inactive monomers for the assembly of active Cre tetramer.

To make it easy to introduce *rpb1* variants into this system, we made a yeast strain (GRY3258) with the *P_HO_-cre-Y324C* substrate, the *his3-AI2floxINV* reporter, and the *rpb1-natMX* deletion complemented by *RPB1* on a *URA3* 2-micron based plasmid (pJS725). As above, this makes it possible to substitute in *rpb1* variants on a *LEU2* based vector by plasmid shuffling, selecting against the *URA3 RPB1* with 5-FOA [51]. Figure 2B illustrates the clear distinction between *RPB1* and the transcription fidelity mutant *rpb1-E1103G* in the number of His^+^ papillae with the *his3-AIloxINV* reporter and the *P_HO_-cre-Y324C* substrate. The increase is dependent on the *cre-Y324C* gene. Similarly, *rpb1-E1230K*, which blocks TFIIS function, gives a clearly elevated signal in this assay.

To identify additional *rpb1* alleles that cause reduced transcription fidelity *in vivo*, we screened a mutagenized pool of a *LEU2 CEN* based plasmid carrying *RPB1* (pJS757). The pool was transformed into GRY3258 selecting Leu^+^ and colonies picked and arrayed as patches. The patches were replica plated to FOA plates to select against the *URA3 RPB1* plasmid. From the FOA plates the patches were replica plated to SC-His media to detect patches with elevated levels of Cre-mediated His^+^ papillae. Mutant candidates were struck for single colonies, and retested as patches. The *LEU2* based plasmid from those that repeated the elevated level of His^+^ papillae was recovered into *E. coli* and retransformed into GRY3258 to confirm that the mutation causing the elevated Cre activity was on the plasmid. Those that passed that test were sequenced to identify the *rpb1* mutation responsible for the transcription infidelity phenotype. In all, over 12,000 transformants were tested from which eight *rpb1* variants were identified. These included re-isolating *rpb1-E1103G*, plus seven new alleles: *rpb1-G823S*, *-A1076T*, *-A1076V*, *-M1079I(T1548I)*, *-K1132E*, *-T1141I(G888D,I1237T)*, and *-S1229F*. The phenotype of the *rpb1-T1141I(G888D,I1237T)* variant was shown to be a consequence of the T1141I substitution by testing variants with each of the separate mutations. Patches demonstrating the phenotypes of these variants are shown in Figure 2B. In addition, we show results for an allele *rpb1-T1113P*, isolated from a screen for alleles that elevate transcription slippage [15].

Quantification of the increase in transcription errors was accomplished by measuring the frequency of His^+^ cells as determined by growth on medium lacking histidine (SC-His) normalized to growth on rich media. The mean frequency from 8 or more cultures was determined for each strain and shows that the mutations identified by our screen increase the frequency of cells with the His^+^ phenotype 7–50 fold compared to the wild type strain (Figure 2C).

### *In vitro* measurement of transcription fidelity

In the *in vivo* screen, increase of the frequency of His^+^ papillae by the mutations in *RPB1* can be indirect. For instance, it could be caused by the increase of *cre* expression because of an increase in initiation by the mutant Pol II. It is noteworthy that the His^+^ frequency is much higher in the *P_HO_*-*cre* system than in the *P_GAL1_-cre* system for the *rpb1-E1103G* allele. In contrast the His+ frequencies for the *rpb1-E1230K* allele are similar in the two systems. Whether this reflects a differential level of sensitivity for these promoters to these defects has not been determined. Furthermore, a higher net frequency of error-containing RNA may be caused not only by the higher frequency of misincorporation (as in the *rpb-E1103G* mutant), but also by higher efficiency of mismatch extension and/or on lower efficiency of RNA editing (like in a *DST1*-deficient strain or in the *rpb1-E1230K* mutant, defective in TFIIS binding) [52]. Below we describe the results of three *in vitro* tests designed to address the biochemical basis for the low-fidelity phenotype of the alleles identified in the genetic screen: 1) the ability of Pol II to select cognate NTP, 2) the capacity to extend a mismatch, and 3) the ability to remove misincorporated NMP by TFIIS-mediated editing.

### Competition fidelity assay

**Figure 3:**
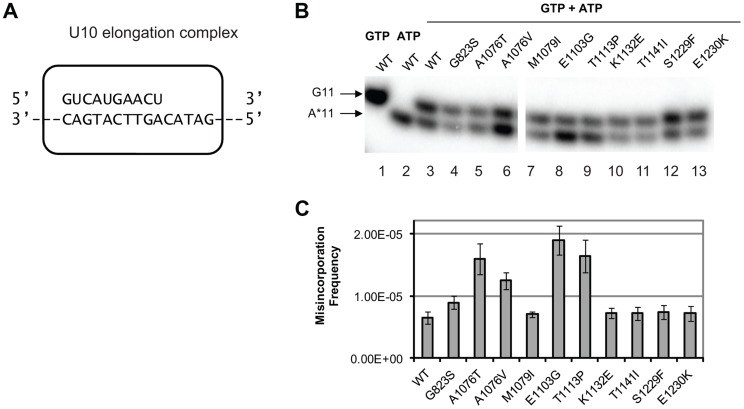
Cognate NTP selection by Pol II variants from the strains with fidelity defects. (**A**) Schematic representation of the elongation complex used for *in vitro* characterization of Pol II. The sequences of nascent RNA in the starting U10 elongation complex and a part of the 57-nt template DNA strand are shown. Pol II is depicted as a rectangular outline with rounded corners. The rest of the template DNA strand and the fully complementary non-template DNA strand are omitted for clarity of presentation. (**B**) Products of cognate and non-cognate incorporation. The TECs were incubated with 20 nM cognate GTP, 1 mM non-cognate ATP or the mix of 20 nM GTP and 1 mM ATP for 5 min. Arrows indicate the products of GMP incorporation and AMP misincorporation (G11 and A*11, respectively). (**C**) ratios of G11 to A*11 products obtained in the presence of 20 nM GTP and 0.5, 1 and 1.5 mM ATP were quantified, normalized to [GTP]/[ATP], and averaged. The error bars show standard deviation.

To determine whether any of the new alleles of *rpb1* have direct impact on cognate NTP selection, we tested *in vitro* the level of misincorporation errors by the Pol II variants. Misincorporation rates and transcription fidelity may depend on a sequence context [4]. Therefore, we employed a DNA corresponding to the region surrounding codon 324 (TGT) in the *cre-Y324C* gene as the template for the assembly of the promoter- and factor-independent Pol II elongation complexes [54] used in these fidelity assays. The elongation complex (U10) was stalled before incorporating AMP and the frequency of GMP to AMP transition error was directly measured using a “competition” assay for transcription fidelity [20], which provides fidelity values nearly identical to those obtained by conventional fidelity assay for the wild type and mutant variants of Pol II [20], [55]. A similar assay has been described for T7 DNA polymerase [56]. The competition assay employs differential mobility in denaturing polyacrylamide gels of short RNA species of the same length, but different composition to distinguish cognate from misincorporation events. To determine the frequency of misincorporation, U10 (schematically depicted in Figure 3A) was incubated with a mix of GTP in a low (20 nM) concentration and different higher (0.5, 0.75 and 1.5 mM) concentrations of ATP. In these conditions, the reaction products of both cognate (GMP) and non-cognate (AMP) incorporation were separated (Figure 3B, lanes 3–13) and quantified (Figure 3C). To determine the misincorporation frequency of AMP in place of GMP in the presence of equal concentrations of cognate and non-cognate substrates, the ratio of non-cognate to cognate products was normalized to (divided by) the ratio of non-cognate (ATP) to cognate (GTP) substrate concentrations. The resulting misincorporation frequency was similar for different concentrations of ATP tested suggesting that it faithfully reflects fidelity of substrate selection in this position, independent of the substrate concentration. This assay is particularly useful way to qualitatively compare the relative misincorporation of various alleles. It is clear that Pol II carrying the *rpb1-E1103G* mutation misincorporates more frequently than wild type Pol II (Figure 3B, lane 8), consistent with its previously reported defect in NTP selection [19]. The *rpb1-A1076T*, *A1076V*, and *T1113P* substitutions also noticeably increase the frequency of misincorporation (lanes 5, 6, and 9). The *RPB1-A1076* residue is located in the trigger loop, a mobile element of the catalytic subunit implicated in transcription fidelity maintenance by genetic and biochemical analyses [3], [19]. In contrast, mutations located in the TFIIS-binding domain (*rpb1-K1132E*, *rpb1-T1141I* and *rpb1-S1229F*) and one mutation in the trigger loop (*rpb1-M1079I*) do not appear to change GMP to AMP transition frequency *in vitro*.

### Mispaired base extension assay

**Figure 4:**
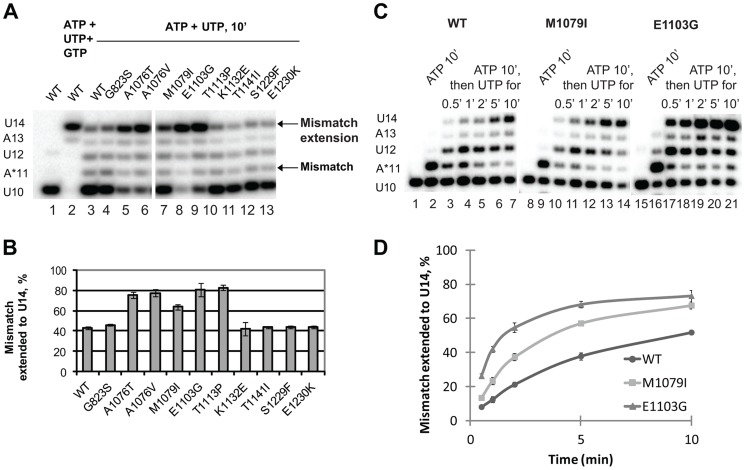
Mismatch extension by Pol II variants carrying mutations in Rpb1. (**A**) Products of simultaneous misincorporation and mismatch extension. The U10 complex was incubated with a mixture of 1 mM ATP and 0.5 mM UTP, or 1 mM ATP, 0.5 mM UTP and 0.5 mM GTP where indicated. The original U10 RNA, A*11 mismatch and mismatch extension products are indicated at left. The arrows at right mark the mismatch (A*11) and mismatch extension products. (**B**) the fraction of U14 mismatch extension product from the sum of A11, U12, A13 and U14 products was quantified; results of three independent experiments were averaged. The error bars show standard deviation. (**C**) Products of consecutive misincorporation and mismatch extension. U10 complexes were incubated in 1 mM ATP for 10 minutes to obtain complexes containing an RNA with a 3′ mismatch (A*11). A sample of A*11 complex for each Pol II variant was taken, and 0.5 mM UTP was added to initiate extension of the mismatch. The extension products were analyzed at the indicated times after the addition of UTP. RNA A*11 and extension products are indicated at left. (**D**) Mismatch extension was quantified as in (B) and plotted versus time. The error bars show standard deviation of three independent experiments.

Next, we tested whether the newly identified *rpb1* alleles alter the ability of Pol II to extend the mismatch by incubating the complex with 1 mM non-complementary ATP and 0.5 mM next cognate UTP to allow misincorporation and mismatch extension. Under these conditions, wild type Pol II does not easily extend the A11 mismatch and pauses at the U12 and A13 positions (Figure 4A, lane 3). Apparently, the mismatch continues to present an obstacle to efficient transcript elongation, consistent with the recent report by Knippa and Peterson [24]. Inefficient mismatch extension, similar to wild type Pol II was observed in the Pol II variants carrying mutations in the TFIIS binding site (*rpb1-K1132E*, *rpb1-T1141I*, *rpb1-S1229F* and *rpb1-E1230K*) (Figure 4A, lanes 10–13). In contrast, mutations in the residues located in the trigger loop (*rpb1-A1076T*, *rpb1-A1076V* and *rpb1-M1079I*) or next to the base of the trigger loop (*rpb1-E1103G* and *rpb1-T1113P*) significantly promote mismatch extension.

The experimental setup described above was chosen because it best reflects the situation *in vivo* when misincorporation occurs in the presence of the next (cognate) NTPs, and the newly formed mismatch can be immediately extended. When the mismatch is pre-formed before the next cognate substrate is provided, Pol II has more time to backtrack, which might artificially decrease the fraction (if backtracking is irreversible) or the rate (if backtracking is reversible) of the mismatch extension. However, if misincorporation is much slower than mismatch extension, the true rate of the latter is difficult to assess in this particular experimental setup. Therefore, mismatch extension by M1079I and E1103G Pol II variants has been assayed in a different setup, when misincorporation was allowed to proceed for 10 min before UTP was added to extend the mismatch (Figure 4C). Quantitative analyses of these data (Figure 4D) confirm our conclusion that mutations in the trigger loop promote mismatch extension.

The enhanced mismatch extension by *rpb1-M1079I* mutant, which does not display increased frequency of misincorporation, provides an explanation for the identification of this allele as error-prone in the *in vivo* screen. Evidently, relatively fast mismatch extension interferes with the post-incorporation error removal by TFIIS-mediated cleavage or the proteolytic degradation of irreversibly arrested Pol II, thus increasing the fraction of the full-length transcripts containing the error. The *in vivo* and *in vitro* properties of Pol II carrying the *rpb1-M1079I* substitution provide direct experimental evidence that recognition of incorporated mismatches by core Pol II significantly contributes to fidelity maintenance.

### TFIIS-dependent editing

**Figure 5:**
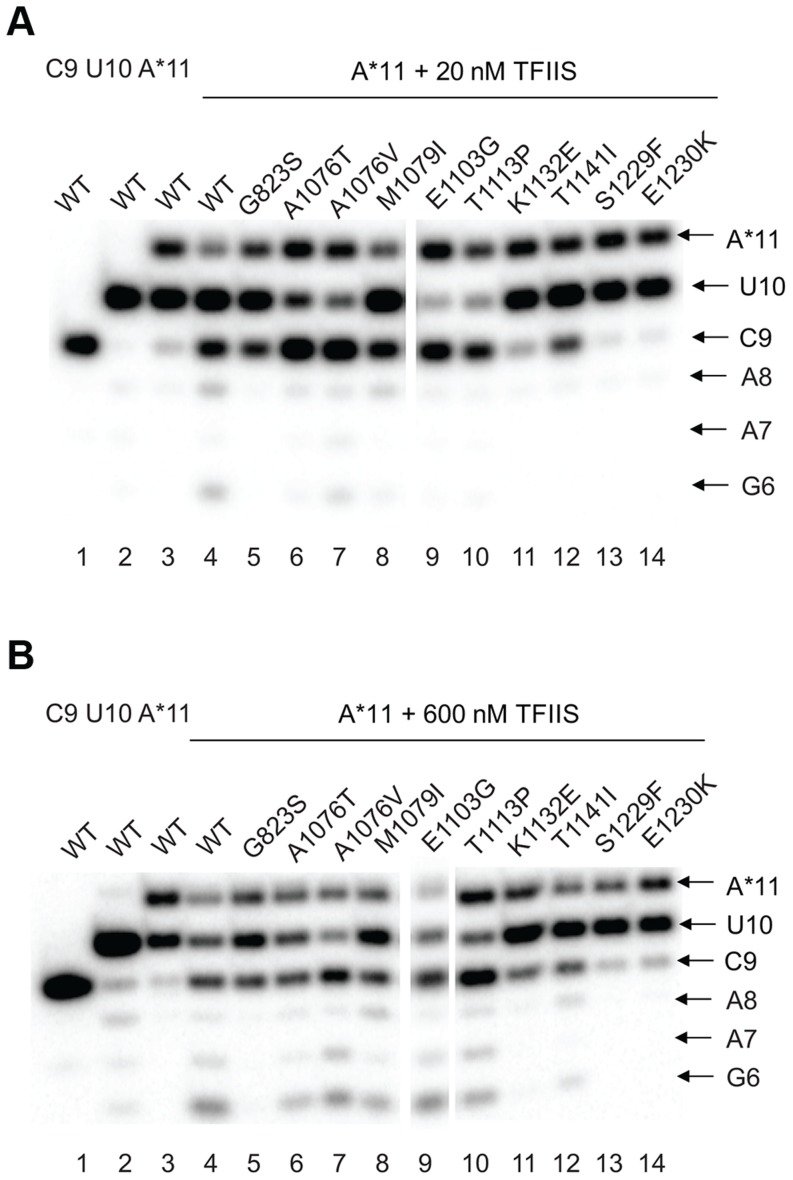
*rpb1* mutations decreasing transcription fidelity *in vivo* interfere with TFIIS-dependent error editing. A*11 complex was obtained as described in Methods and incubated with 20 nM (**A**) or 600 nM (**B**) TFIIS for 1 min. The products of TFIIS-induced RNA cleavage (C9, A8, A7, and G6) are indicated at right.

The effect of the newly identified mutations on interaction of Pol II with TFIIS has been tested by treating the A*11 elongation complex carrying the 3′-end mismatch with TFIIS, and observing the extent of 3′RNA cleavage (Figure 5). The 3′ mismatched AMP and the complementary penultimate UMP are removed after one minute incubation with TFIIS in the major fraction of the wild type complexes, resulting in appearance of a 9-nt RNA (C9). Note that the C9 product was barely detectable in the initial A*11 elongation complexes (lane 3 in Fig. 5 A and B). The shorter RNA cleavage products (A8, A7, and G6) are also detected, especially after incubation with higher concentration (600 nM) TFIIS (Fig. 5B, lane 4). The shorter RNA cleavage products (A8, A7, and G6) are also detected, especially after incubation with higher concentration (600 nM) TFIIS (Figure 5B, lane 4). The appearance of the 5′ cleavage products as short as 6 nt is somewhat unexpected, considering that the RNA-DNA hybrid in Pol II elongation complex is 8-bp long [57]. Nevertheless, we use appearance of the short cleavage products, along with the major C9 product, to judge the efficiency of TFIIS-induced RNA cleavage.

As expected, substitutions in the TFIIS binding site (*rpb1-S1229F* and *rpb1-E1230K*) dramatically decreased Pol II susceptibility to TFIIS-induced mismatch removal. The C9 cleavage product appears only when the elongation complexes are treated with the higher concentration (600 nM) TFIIS, and no shorter products are detected (compare lane 4 with lanes 13 and 14 in Figure 5A&B,). A less pronounced, but still substantial decrease in the sensitivity to TFIIS is observed for *rpb1-G823S*, *K1132E*, and *rpb1-T1141I* mutants (lanes 5, 11, and 12). It is likely that K1132 and T1141 substitutions directly reduce binding of TFIIS. The G823S substitution in the bridge helix may alter TFIIS binding, but alternatively could be involved in TFIIS-induced transcript cleavage, and/or affect Pol II backtracking. The effect of T1141I substitution was weaker than the effects of the other three substitutions in the TFIIS binding site (note the presence of the short cleavage products in Figure 5B, lane 11). It is interesting that Rpb1-G823S, which shows a decreased sensitivity to TFIIS similar to Rpb1-K1132E substitution, also slightly promotes misincorporation and mismatch extension *in vitro* (Figures 3 and 4). All other mutants tested for susceptibility to TFIIS were similar to wild type Pol II (Note the A8, A7 and G6 products in Figure 5B, lanes 4 and 6–10).

## Discussion

We present here a solution to problematic issues about the measurement of transcription fidelity *in vivo*. Previous results suggested that G to A transitions represent the major class of errors generated by yeast Pol II [19] and by the *E. coli* RNA polymerase *in vitro* and in living cells [58]. Using that information, we developed a second generation assay specific for the detection of G to A transcription errors. The assay is based on a mutation of the active site TAT tyrosine codon of the Cre recombinase to TGT. Rare Pol II errors that restore UAU to that position allow the production of active Cre protein whose activity is detected by inversion of a 4.8 kb interval to restore a selectable phenotype (His^+^). The requirement that each of the four subunits of the Cre tetramer is active eliminates the background from translation errors. The assay converts a transient Cre activity into a stable genetic change allowing the detection of Cre activity at frequencies less than 10^−5^. This sets a lower bound on net transcription error rates, but it is likely that it underestimates the real level of uncorrected G to A substitutions during transcription because the efficiency of assembling an active Cre tetramer and the efficiency of the recognition and recombination of the lox sites in the reporter gene are unknown.

### Identification of Rpb1 regions involved in control of fidelity *in vivo*

**Figure 6:**
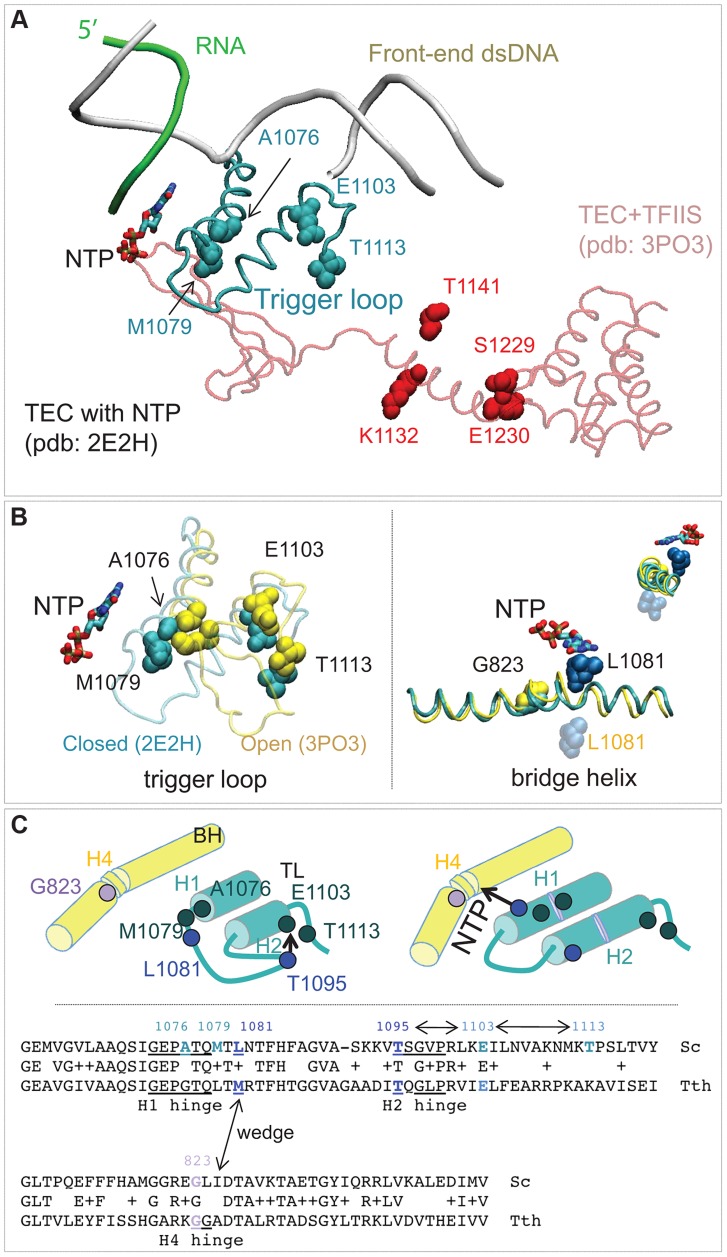
Rpb1 domains involved in control of transcription fidelity. (**A**) Clusters of residues implicated in transcriptioin fidelity control by genetic screening and biochemical analyses. RNA, DNA, substrate NTP, and the closed trigger loop (TL) are from pdb 2E2H; this structure is aligned with the X-ray structure of TFIIS taken from the backtracked Pol II/TFIIS complex (pdb 3PO3, shown in light red). (**B**) Location of Rpb1-A1076, M1079, E1103 and T1113 residues between the open (yellow; pdb 3PO3) and the closed (cyan; pdb 2E2H) states of the TL (*left panel*). *On the right:* Rpb1-G823 (yellow) is located next to the site of the bridge helix bending. The long-distance movement of L1081 residue of the TL associated with the bridge helix bending and the loop opening is shown. The upper right corner shows a view along the long axis of the bridge helix. (**C**) The cartoon illustrates the major conformational changes near the active site associated with the TL closure and opening and identifies the four hinges in the trigger loop and bridge helix. *At the bottom:* an alignment of the TL and the bridge helix amino acid sequences from the yeast Pol II and *T. thermophilus* RNAP with all hinges and L1081 residue highlighted. The TL and bridge helix crosstalks are indicated by double-headed arrows.

The random mutagenesis of the entire *RPB1 (RPO21)* gene yielded three groups of amino acid residues that are highly clustered in the X-ray structure of Pol II [18]. The *rpb1-A1076T/V*, *rpb1-M1079I*, and *rpb1-E1103G* mutations alter the trigger loop, a domain of Pol II that closes over the active site and has been demonstrated to influence fidelity *in vitro* [3], [19]. The *rpb1-T1113P* mutation targets a residue in the immediate vicinity of E1103 (Figure 6A). The *rpb1-G823S* allele (Figure 6B, right panel) alters the bridge helix, a domain that interacts with the active site and with the trigger loop. Substitutions in the bridge helix of *E. coli* RNA polymerase have been demonstrated to reduce the fidelity of transcription *in vitro* [59]. Notably, the changes identified in this work directly target the flexible hinge regions of the trigger loop and the bridge helix that were identified by molecular dynamics simulations based on X-ray crystallography and confirmed by mutational analysis of yeast Pol II [20]. The hinges, H1 and H2 in the trigger loop and H3 and H4 in the bridge helix, according to the nomenclature from [20] (Figure 6C) undergo conformational changes associated with NTP binding, sequestration, catalysis and translocation. The trigger loop and bridge helix residues forming the hinges have been implicated in transcription control [19], [55], [59], [60].

Another cluster of mutations alters the TFIIS binding site (Figure 4A, residues shown in red) [61]. The *rpb1-S1229F* mutation likely reflects a defect in the interaction of Rpb1 and TFIIS, similar to the well characterized *rpb1-E1230K* allele [52]. The *rpb1-K1132E* and *rpb1-T1141I* substitutions also alter positions close to where TFIIS binds [61]. These mutations may impair TFIIS-dependent error editing by decreasing TFIIS binding, similar to mutations of E1230 and S1229. Alternatively, they might affect the backtracking capabilities of Pol II, a mechanism related to Pol II translocation and likely affected by the G823S substitution. The precise characterization of TFIIS cleavage mechanisms affected by mutations identified here is beyond the scope of this work. Most importantly, our results represent a direct demonstration of proofreading function of TFIIS in living cells. Although there is firm evidence that TFIIS plays a major role in correction of transcription errors *in vitro*, several attempts to demonstrate the similar activity *in vivo* were inconclusive [10], [11]. Our results are consistent with those by Koyama and co-workers showing a major contribution of TFIIS to Pol II fidelity in yeast [12], [21].

### Pol II fidelity is under multilayer control *in vivo*

Our *in vivo* approach combined with the biochemical *in vitro* validation revealed at least three intrinsic and one factor-dependent mechanism for faithful transcription in living cells. The TFIIS-dependent mechanism has been discussed above. The first intrinsic mechanism includes regulation of the trigger loop movement. Interaction of Rpb1 E1103 residue with the H2 hinge (residues 1095–1099) of the trigger loop has been previously proposed to delay the trigger loop closure thus slowing down transcription elongation and supporting fidelity maintenance [3], [19], [23], [62]. The observation that *rpb1-T1113P* substitution renders transcription error-prone indicates that the T1113 residue plays the same or similar role as E1103. Notably, the recent molecular dynamic simulations of the Pol II trigger loop opening and closure revealed the potential interaction of T1113 with the trigger loop predicting that substitutions of T1113 residue should increase transcription elongation rate [63]. Our work provides direct proof that the mechanism dependent on E1103 and T1113 interaction with the trigger loop acts *in vivo*.

The second potential mechanism includes H1 hinge of the trigger loop (Rpb1-A1076/M1079) and H4 hinge of the bridge helix (Rpb1-G823) that may crosstalk through the adjacent L1081 (wedging) residue of the trigger loop [64] (Figure 6B&C). Because conformational changes of the bridge helix and the trigger loop are implicated in translocation [64]–[66] our identification of error-prone mutants in the mobile hinges of these two structural elements suggests a possible connection of the Pol II translocation cycle and cognate NTP selection. The mechanism of this link remains to be established.

The third intrinsic mechanism revealed by our work does not immediately follow from the extensive *in vitro* studies of transcription fidelity. It involves inhibition of the mismatch extension with the next cognate NMP. This mechanism, apparently mediated by the trigger loop and bridge helix, promotes Pol II pausing or arrest after misincorporation. Our finding that several mutations selected in the Cre-based genetic screen promote mismatch extension *in vitro* strongly argues that slow extension of a mismatch in the nascent RNA plays a major role in faithful transcription in living cells by allowing correction to occur. The *rpb1-M1079I* allele identified here appears of special interest, because it does not affect NTP selection by Pol II, but clearly promotes mismatch extension. Because the net frequency of *in vivo* transcription error occurrence correlates with the propensity of *E. coli* RNA polymerase to backtrack within a given sequence context [58], we are currently investigating the effect of *rpb1-M1079I* substitution on the mismatch-induced transcription arrest.

The slow mismatch extension may enable correction of the error by TFIIS-mediated transcript cleavage [27]. The arrest may also provide time for elimination of the flawed transcript by ubiquitin-mediated proteolytic degradation of Pol II [67]. One can imagine *rpb1* alleles that impair accuracy in nucleotide selection, but, due to a reduced transcription elongation rate [3], [20], [60] provide increased opportunity to detect and remove misincorporated NMPs. Thus, slow elongation that results in poor mismatch extension could reduce production of the full-length error-containing Cre mRNA, counter-acting the defect in substrate selection. Accordingly, faster elongation might further decrease overall fidelity of the mutants with impaired NTP selectivity, such as *rpb1-A1076T, A1076V, E1103G* and *T1113P* [19].

In conclusion, we developed a reliable experimental approach to monitor transcription fidelity *in vivo*. Using this tool we will characterize the role of other core Pol II subunits, as well as known transcription elongation factors, such as TFIIF, Spt4/5 and Spt6 in transcription fidelity maintenance. This methodology will allow for isolation of mutants affecting transcription fidelity and thus will promote identification of new genes and mechanisms related to the accuracy of transcription. These experiments also highlight the complications associated with assigning a specific phenotype solely to the fidelity of transcription. The *rpb1-E1103G* mutation increases the transcription error rate. When combined with a defect in TFIIS (*dst1Δ*), which corrects transcription misincorporation errors, the double mutant is dead. It is tempting to conclude that the death is the direct result of an error catastrophe of too many transcription errors resulting in too many incorrect RNAs and defective proteins encoded by them. However, TFIIS also has a role in transcription initiation at some promoters and the lethality of the double mutant could reflect some more complicated interplay of the properties of the defective RNA polymerase and the changes in the transcriptome caused by the TFIIS defect. Similarly, it was possible that an increase in the frequency of His^+^ cells in our genetic assay reflected changes in the expression level of the *cre* reporter genes caused by a change in the transcriptome unrelated to reduced fidelity. A recent survey of changes in the yeast transcriptome caused by RNA polymerase variants further emphasizes this biological feature [28]. They found changes in transcription start sites, altered relative transcript levels, and changes in splicing efficiency. Thus, it was important to include a biochemical characterization of the mutant polymerases to see if there is a fidelity defect. We characterized misincorporation, mispaired base extension, and mispaired base removal and found defects in one or more of these processes in each of the mutants. These mutants provide an opportunity to elucidate the cellular consequences of error prone transcription. The work described here is a step to understanding the biological role of faithful information transfer from DNA to RNA.

## Methods

### Plasmids

See Table 1. pJS757 is a *LEU2*-based *CEN* vector carrying the *RPB1* ORF, as well as 594 bases upstream and 501 bases downstream of the ORF [16]. pJS725 and pJDI220 are *URA3*-based 2-micron circle plasmids with the same *RPB1* insertion as pJS757. *RPB1* was randomly mutagenized by passing a pool of plasmid pJS757 twice through the mutator (*mutS mutD mutT*) *E. coli* strain XL-1 Red (Stratagene).

**Table 1:** Plasmids and yeast strains.

Plasmids:		
pJDI220	*RPB1*	*URA3* 2-micron circle plasmid
pJS670	*RPB1*	*LEU2 CEN* plasmid
pJS725	*RPB1*	*URA3* 2-micron circle plasmid
pJS757	*RPB1*	*LEU2 CEN* plasmid
pJS781	*rpb1-E1103G*	*LEU2 CEN* plasmid
pJS932	*rpb1-E1230K* (*rpo21-24*)	*LEU2 CEN* plasmid
pJS1006	*rpb1-A1076T*	*LEU2 CEN* plasmid
pJS1007	*rpb1-G823S*	*LEU2 CEN* plasmid
pJS1008	*rpb1-S1229F*	*LEU2 CEN* plasmid
pJS1009	*rpb1-A1076V*	*LEU2 CEN* plasmid
pJS1010	*rpb1-M1079I, T1548I*	*LEU2 CEN* plasmid
pJS1025	*rpb1-K1132E*	*LEU2 CEN* plasmid
pJS1184	*rpb1-T1141I*	*LEU2 CEN* plasmid
pJS1212	*rpb1-T1113P*	*LEU2 CEN* plasmid

Yeast Strains:		
GRY2852	*MAT*a, *his3-AI2floxINV* (*hygMX*, *zeoMX*), *leu2-Δ0, met15-Δ0, ura3-Δ0*,	
	*bud5::P_GAL1_-cre-WT, rpb1::natMX*,	
	[pJDI220 (*RPB1 URA3*)].	
GRY2855	*MAT*a, *his3-AI2floxINV* (*hygMX*, *zeoMX*), *leu2-Δ0, met15-Δ0, ura3-Δ0*,	
	*bud5::P_GAL1_-cre-Y324C, rpb1::natMX*,	
	[pJDI220 (*RPB1, URA3*)].	
GRY3175	*MAT*α, *his3, leu2-Δ0, ura3, his3, lys2-Δ0, trp1-361, pep4::HIS3*, *prb1-Δ1.6R*	
	*rpb1::kanMX, RPB3::6XHis*	
	[pJS725 (*RPB1 URA3*)].	
GRY3258	*MAT*α, *his3-AI2floxINV* (*hygMX*, *zeoMX*), *leu2-Δ0, ura3-Δ0, rpb1::natMX*,	
	*P_HO_-cre-Y324C*,	
	[pJDI220 (*RPB1, URA3*)].	
GRY3284	*MAT*α, *his3-AI2floxINV* (*hygMX*, *zeoMX*), *leu2-Δ0, lys2-Δ0, ura3-Δ0*,	
	*rpb1::natMX, P_HO_-cre-Y324C*,	
	[pJS781 (*rpb1-E1103G, LEU2*)].	
GRY3739	*MAT*a, *his3-AI2floxINV* (*hygMX*, *zeoMX*), *leu2-Δ0, met15-Δ0, ura3-Δ0*,	
	*bud5::P_GAL1_-cre-Y324C, rpb1::natMX*,	
	[pJS670 (*LEU2, RPB1*)].	
GRY3740	*MAT*a, *his3-AI2floxINV* (*hygMX*, *zeoMX*), *leu2-Δ0, met15-Δ0, ura3-Δ0*,	
	*bud5::P_GAL1_, rpb1::natMX*,	
	[pJS670 (*LEU2, RPB1*)].	
GRY3742	*MAT*a, *his3-AI2floxINV* (*hygMX*, *zeoMX*), *leu2-Δ0, met15-Δ0, ura3-Δ0*,	
	*bud5::P_GAL1_-cre-Y324C dst1::natMX*	
GRY3743	*MAT*a, *his3-AI2floxINV* (*hygMX*, *zeoMX*), *leu2-Δ0, met15-Δ0, ura3-Δ0, trp1*,	
	*bud5::P_GAL1_-cre-Y324C, rpb9::kanMX*	

### Yeast strains

The yeast strains are related to the BY4741 and BY4742 [68] See Table 1.

### Cre expression

The *cre* gene used in these experiments has a seven amino acid nuclear localization site from the large SV40 T-antigen added at the N-terminus [69]. The *P_GAL1_-cre* gene was made by fusing the *NLS-cre* ORF to the *P_GAL1_* promoter inserted into the *BUD5* gene at an autochthonous *EcoR1* site. The *P_HO_-cre* gene was made by overlap PCR and integrated at the normal position for *HO*. The insertions were made by selecting for replacement of *URA3*, previously inserted at those locations.

### Reporters for Cre activity

*Direct repeat of loxP:* The *his3-AI2floxkanMX* reporter (Figure 1B, construct (iii)) has an artificial intron inserted into *HIS3* at an *MscI* site in codon 70. The 1710 base insertion includes two loxP sites in direct orientation flanking the *kanMX* cassette. *kanMX* is in the same orientation as *HIS3* so that the terminator blocks transcription through the rest of *HIS3* resulting in cells that are His^−^. The Cre recombinase can remove the 1502 bases including *kanMX* plus one loxP resulting in a spliceable intron (*HIS3-AI2lox* Figure 1B, (ii)) and cells that are His^+^. The *ade6-AI2floxkanMX* reporter has the same artificial intron inserted after the tenth codon of *ADE6* causing cells to be *ade6^−^*, but Cre recombinase can remove *kanMX* and make a functional *ADE6-AI2lox* gene. *Inverted repeat of loxP:* The *his3-AI2floxINV* substrate (Figure 1B, (iv)) was constructed on chromosome XV at the *HIS3* locus and surrounding region. This reporter contains the N-terminal portion of *HIS3-AI2* and the splice donor part of the artificial intron ending at the loxP site and is marked by hygromycin resistance (*hygMX*). A lox site, the splice acceptor portion of the intron and the C-terminal portion of the *HIS3-AI2*, marked by zeomycin resistance (*zeoMX*), was inserted in inverted orientation 682 bases downstream of the *GEP3* gene (2.8 kb beyond *HIS3*). Cre-directed recombination inverts the floxed DNA (including the *DED1* gene) creating the functional *HIS3-AI2lox* gene (Figure 1B-v). Hygromycin (*hygMX*) is from pAG32 [70] and Zeocin (*zeoMX*) is from pHybLex/Zeo (Invitrogen).

### *In vitro* transcription assays

Pol II variants carrying wild type or mutant Rpb1 were introduced into a protease-deficient yeast strain GRY3175 by plasmid shuffle. Hexahistidine-tagged Rpb3 [57] was used to pull down Pol II from the whole cell lysate essentially as described [54]. Specifically, cells from 3–4 ml of stationary (2–3 days) culture were pelleted, washed once with cold water and resuspended in lysis buffer (150 mM Tris–acetate, pH 7.9, 50 mM potassium acetate, 5 mM MgCl_2_, 10 µM ZnCl_2_, 2 mM 2-mercaptoethanol, 0.5 mM EDTA and protease inhibitors). The cells were disrupted using a Precellys homogenizer according to manufacturer's instructions, the lysate was removed from the glass beads, and KCl was added to 1M final concentration. The debris was precipitated for 15 min at 14,000 rpm in Eppendorf table-top microcentrifuge at 4°C. The supernatant was added to 50 µl of Ni-NTA agarose pre-washed with transcription buffer (TB) containing 20 mM Tris-HCl, pH 7.9, 5 mM MgCl_2_, 10 µM ZnCl_2_, 2 mM 2-mercaptoethanol and 1000 mM KCl (TB1000). Pol II was immobilized on the beads for 30–50 min at 4°C, and the beads were extensively washed with TB1000 and TB40 (TB with 40 mM KCl). The elongation complex was assembled and purified with the immobilized Pol II exactly as described [54] using the following oligonucleotides: Cre RNA C9: 5′ GUC AUG AAC 3′(phosphorylated with T4 polynucleotide kinase in the presence of γ-P32-labeled ATP); NDS-Cre-Cys (Non Transcribed DNA Strand) 5′GGCTGGACCAATGTAAATATTGTCATGAAC TGTATCCGTAA CCTGGATAGTGAAACA 3′; and TDS-Cre-Cys (Transcribed DNA Strand) which is an exact complement of NDS (Cre-Cys). All NTPs used for walking and misincorporation assays were additionally purified [19]. The elongation complex containing 10-nt RNA (U10) was obtained by 5 min incubation of the assembled and purified complex with 5 µM UTP and washed 4 times with 1 ml of TB40. For the TFIIS sensitivity assay, mismatched A11 elongation complex was obtained by 5 min incubation of U10 with 1 mM ATP, purified by 4 washes with TB40, eluted from the beads with 100 mM imidazole in the presence of 0.2 mg/ml acetylated BSA, diluted 10-fold with TB40 to decrease imidazole concentration and concentrated using Microcon AmiconUltra (Millipore) concentrator to 30 µl. Reactions were stopped by addition of equal volume of gel loading buffer containing 10 M urea and 50 mM EDTA. The reaction products were resolved in 20% polyacrylamide gels (19∶1) in the presence of 1× TBE and 7M urea. To resolve the two 11-nt products of cognate GMP and mismatched AMP incorporation in the competition fidelity assay, the 4-mm thick, 20×40 cm gels were used, and electrophoresis has been performed in 1× TBE at constant power of 65 W until the bromphenol blue tracing dye was within 3 cm of the bottom of the gel. For other experiments the shorter 20×20 cm gels were used and the electrophoresis was performed at 50 W constant power.
